# Induction chemotherapy followed by concurrent chemoradiotherapy is benefit for advanced stage nasopharyngeal carcinoma with different nonkeratinizing carcinoma subtypes

**DOI:** 10.1038/s41598-018-31050-z

**Published:** 2018-09-06

**Authors:** Jian Zang, Chen Li, Man Xu, Wanni Xu, Xiaowei Kang, Jianhua Wang, Shanquan Luo, Mei Shi

**Affiliations:** 1 Department of Radiation Oncology, xijing Hospital, Fourth Military Medical University, Xi’an, Shanxi China; 2 Department of Health Statistics, Faculty of Preventative Medicine, Fourth Military Medical University, Xi’an, Shanxi China; 3 Department of radiology, xijing hospital, Fourth Military Medical University, Xi’an, Shanxi China; 4 State key laboratory of cancer biology and department of pathology, Fourth Military Medical University, Xi’an, Shanxi China

## Abstract

Given the potentially distinctive histological variations in northwest of China, the aim of current study was to compare the efficacy of induction chemotherapy plus concurrent chemoradiotherapy (IC + CCRT) with concurrent chemoradiotherapy (CCRT) in nasopharyngeal carcinoma (NPC) patients with different histological types. A total of 301 patients were included in this study. Patients were classified in two cohorts according to the 2005 WHO World Health Organization histological classification: WHO type IIa group and WHO type IIb group. The Kaplan-Meier method was used to detect the efficacy between IC + CCRT and CCRT in two WHO types cohorts. Propensity score matching method was adopted to balance the baseline covariate and eliminate potential selection bias. On propensity matched analyses, IC + CCRT was found to produce better 3-year DMFS and OS than CCRT in WHO type IIa cohort (DMFS, 76.2% vs. 42.2%, p = 0.029; OS, 78.3% vs. 65.5%, p = 0.027). For WHO type IIb cohort, IC + CCRT was associated with a better 3-year OS (87.4% vs. 77.9%, p = 0.029) and a trend of better 3-year DMFS (85.9% vs. 76%, p = 0.162) compared with CCRT. IC + CCRT was benefit for advanced stage nasopharyngeal carcinoma with different nonkeratinizing carcinoma subtypes.

## Introduction

Nasopharyngeal carcinoma (NPC) is an unbalanced geographic distribution disease. The incidence of NPC in endemic area of China is approximately 15–30 for every 100,000 people per year^1^. In China, the highest incidence rate occurs in Southern China, whereas in northwest China, the incidence rate is low. WHO histological type has been identified as a significantly prognostic factor to impact the survival of NPC patients by several epidemiology studies^2,3^. According to the 2005 WHO classification, NPC histological types were classified as keratinizing carcinoma (type I) and nonkeratinizing squamous cell carcinoma (type II). Type II was further classified to two subtypes: differentiation subtype (type IIa) and undifferentiation subtype (type IIb)^4^. Our previous studies reported that NPC in northwest China had higher proportion of WHO type IIa (approximate 30%) than endemic area of China (<5%)^5–7^. Compared with the most common WHO type IIb, several studies showed WHO type IIa contributed to poor overall survival (OS) and distant metastasis-free survival (DMFS) for patients with NPC^5,8,9^. These results implied more intensity treatment modality should be delivered to patients with WHO type IIa.

Concurrent chemoradiotherapy (CCRT) plus adjuvant chemotherapy has become a standard treatment modalities for advanced NPC for many years since it was established by the intergroup 0099^10^. However, the treatment modalities were changing along with the combinations of new radiotherapy technique and drugs. In the era of intensity modulated radiotherapy (IMRT), 5-year local recurrence-free survival (LRFS) of locoregionally advanced stage NPC has achieved more than 90%, whereas 5-year DMFS and OS are limited to70–80%^11^. MAC-NPC meta-analysis suggested induction or adjuvant chemotherapy might be a promising treatment modalities to further improve the DMFS and OS for patients with advanced stage disease^12^. Recently, a phase III randomized study showed that adjuvant cisplatin and fluorouracil (PF) chemotherapy did not improve the treatment outcomes^13^. However, compared with CCRT, induction chemotherapy followed by concurrent chemoradiotherapy (IC + CCRT) was identified to be benefit to improve the 3-year DMFS and OS by another phase III randomized study from endemic region of China^14^. At present, IC + CCRT has been considered as a dominating treatment modality for endemic NPC with clinical advanced stage. However, two important issue remain unsettled for the clinicians from northwest region of China: (1) which is the better choice between IC + CCRT and CCRT for the locoregionally advanced NPC in non-endemic region of China? (2) especially for patients with WHO type IIa, whether IC + CCRT is a better option to improve survival outcomes compared with CCRT alone.

The aim of current study was to compare the efficacy of IC + CCRT with CCRT in patients with different nonkeratinizing carcinoma subtypes from northwest of China. Propensity score matching method was adopted to balance the baseline covariate and eliminate potential selection bias.

## Materials and Methods

### Patients selection

We reviewed 524 cases of histologically proven NPC patients, who received initial treatment at our institute between January 2006 to December 2014. The inclusion criteria were as follow: histologically confirmed non-keratinizing carcinoma of nasopharynx by biopsy; locoregionally advanced stage III-IVB without metastasis; receiving IC + CCRT or CCRT as initial treatment modality; receiving IMRT as definitive radiotherapy; patients’ primary residences limited to the northwest of China. We excluded patients who did not complete the prescribed course of radiotherapy, who developed non-cancer specific death. Ultimately, a total of 301 patients were included for analysis. All methods were carried out in accordance with the guidelines and regulations of ethics committee of Xijing hospital. This study was approved by the ethics committee of XiJing Hospital, Xi’an, China. The ethics committee of our hospital confirmed it was not necessary to obtain informed consent in this study because there were no participants involved during the research process. All research materials were obtained on the base of the computerized patient record system of xijing hospital.

### Clinical staging

The routine staging workup included a complete history and physical examinations, blood work, direct fibreoptic nasopharyngoscopy, imaged by computed tomography (CT) and magnetic resonance imaging (MRI) of head and neck, and chest images, abdominal sonography, and whole body bone scan, as well as positron emission tomography (PET)-CT, if necessary. All patients MRI detail were evaluated by two experienced radiologists. Consensus meetings were conducted to resolve the disagreements. All patients were restaged according to the 7th editions of the International Union against Cancer/American Joint Committee on Cancer (UICC/AJCC) system.

### Histological type

The haematoxylin and eosin stained sections of biopsy material obtained for first diagnosis were retrieved and reviewed by two senior pathologists. The histological type was identified according to the 2005 WHO World Health Organization classification based on the microscopy morphology^4^. The non-keratinizing undifferentiated type (WHO type IIb) was characterized by syncytial-appearing large tumor cells with indistinct cell borders, round to oval vesicular nuclei, and large central nucleoli. The non-keratinizing differentiated type (WHO type II) usually showed cellular stratification and pavementing, often with a plexiform growth, reminiscent of transitional cell carcinoma of the bladder. In the present study, when both types were seen in a specimen, the differentiated component had to constitute more than 50% of the tumor tissue to be qualified as differentiated type.

### Clinical treatment

The treatment planning approaches were described by our previous studies^5,9,15^. The prescribe dose were 72.6 Gy in 33 fractions to the planning target volume (PTV) of gross tumor volume of nasopharynx (GTVnx), 66 to 72.6 Gy to PTV of gross tumor volume of positive lymph nodes (GTVnd), 66 Gy to the entire nasopharynx mucosa, 60 to 63 Gy to PTV of high risk clinical target volume (CTV1), and 50.4 to 56 Gy to PTV of low risk clinical target volume (CTV2). The dose received by each organ at risk (OAR) should be no more than its tolerance^16^.

The neoadjuvant chemotherapy included TP regimen (docetaxel 75 mg/m^2^, cisplatin 75 mg/m^2^), PF regimen (cisplatin 80 mg/m^2^, 5-FU 800–1000 mg/m^2^ days 1 to 5), GP regimen (gemcitabine 1000 mg/m^2^, cisplatin 75 mg/m^2^) and TPF regimen (docetaxel 75 mg/m^2^, cisplatin 75 mg/m^2^, 5-FU 750 mg/m^2^ days1 to 5) every 3 weeks for 2–3 cycles at a 2–3 weeks’ interval before the initial radiotherapy. Concurrent chemotherapy was only consisted of cisplatin (100 mg/m^2^ every 3 weeks or 40 mg/m^2^ weekly).

### Statistical analysis

The endpoints included LRFS, DMFS, progression-free survival (PFS) and OS which were defined as time to first local recurrence and/or distant metastasis. Propensity scores were computed by logistic regression for each patient using the following covariates: age, gender, smoking, drinking, race, blood EBV DNA copies, T category, N category, clinical stage, histological WHO type, tumor volume. Initial propensity matching was conducted with a 1:1 match of IC + CCRT to CCRT. Because the sample of IC + CCRT was almost two times as large as CCRT, another propensity matching was undertaken with two IC + CCRT patients matched to one CCRT patients through a Greedy algorithm with caliper being 0.2 times of standard deviation of logit propensity score. Numerical variable was transformed to categorical variable using interquartile range method if it was not Gaussian distribution, such as tumor volume. Means were compared by the Student’s t test. Categorical variables were compared by the χ^2^ test. The Kaplan-Meier method was used to calculate the accurate rate of endpoints. Because of high distant failure rate in advanced NPC, the prognostic analysis only focused on DMFS and OS. Only the factors which were found to be associated with the endpoints by univariate analyses entered into multivariate Cox proportional hazards regression analysis. The hazard ratio (HR) and its 95% confidence interval (95% CI) were used to indicate the prognostic value of risk factors. A 2-sided p value of less than 0.05 was considered significant. SAS statistical package 9.1.3 (SAS institute USA) and GraphPad Prsim 5.0 (GraphPad Software Inc, USA) were used for all analyses.

## Results

### Patient characteristics

Patient characteristics are presented in Table 1. The patients’ overall median age was 47 years old (range, 18–78 years). Clinical stage IV was the most common stage in this study (72.1%). Besides T stage and N stage, the baseline demographic and clinical characteristics of the treatment groups were well balanced before matching. But the distribution biases were eliminated after matching.

**Table 1:** Clinical characteristics of all patients before and after PSM by treatment.

	Before match		P value	After match		P value
	IC + CCRT N (%)	CCRT N (%)		IC + CCRT	CCRT	
Gender						
Male	164 (76.6)	67 (77)	0.944	93 (81.6)	44 (77.2)	0.498
female	50 (23.4)	20 (23)		23 (18.4)	13 (22.8)	
age						
≤50	135 (63.1)	48 (55.2)	0.202	67 (58.8)	35 (61.4)	0.741
>50	79 (36.9)	39 (44.8)		47 (41.2)	22 (38.6)	
Race						
Ethnic Han	207 (96.7)	86 (98.9)	0.52	108 (94.7)	56 (98.2)	0.495
others	7 (3.3)	1 (1.1)		6 (5.3)	1 (1.8)	
Smoke						
No	104 (48.6)	51 (58.6)	0.115	60 (52.6)	32 (56.1)	0.664
yes	111 (51.4)	36 (41.4)		54 (47.4)	25 (43.9)	
Smoke index						0.736
mean	257.64 ± 25.026	208.28 ± 36.459	0.28	213.24 ± 30.5	195.7 ± 40.0	
Drink						
No	134 (62.6)	62 (71.3)	0.154	74 (64.9)	38 (66.7)	0.82
Yes	80 (37.4)	25 (28.7)		40 (35.1)	19 (33.3)	
AJCC stage						
III	57 (26.6)	27 (31)	0.441	40 (35.1)	20 (35.1)	0.99
IV	157 (73.4)	60 (69)		74 (64.9)	37 (64.9)	
T stage						
T1	11 (5.1)	6 (6.9)	0.021	6 (5.3)	6 (10.5)	0.39
T2	63 (29.4)	13 (14.9)		35 (30.7)	12 (21.1)	
T3	40 (18.7)	13 (14.9)		15 (13.2)	7 (12.3)	
T4	100 (46.7)	55 (63.2)		58 (50.9)	3 (56.1)	
N stage						
N0	5 (2.3)	22 (25.3)	<0.001	0 (0)	0 (0)	0.822
N1	21 (9.8)	13 (14.9)		9 (7.9)	6 (10.5)	
N2	132 (61.7)	47 (54)		96 (84.2)	46 (80.7)	
N3	56 (26.2)	5 (5.7)		9 (7.9)	5 (8.8)	
WHO type						
IIa	63 (29.4)	28 (32.3)	0.638	35 (30.7)	13 (22.8)	0.279
IIb	151 (70.6)	59 (67.8)		79 (69.3)	44 (77.2)	
Tumor volume ml						
Median (range)	40.37 (12.5–224.1)	45.72 (7.5–135.6)		42 (12.5–189.4)	42.1 (7.5–135.6)	
<23.75	58 (27.1)	17 (19.5)	0.435	31 (27.2)	15 (26.3)	0.906
23.75–42.14	53 (24.8)	23 (26.4)		26 (22.8)	14 (24.6)	
42.15–64.67	49 (22.9)	26 (29.9)		30 (26.3)	17 (29.9)	
>64.67	54 (25.2)	21 (24.1)		2 (23.7)	11 (19.3)	
EBV DNA						
<5000 copies/ml	202 (94.4)	79 (90.8)	0.257	109 (95.6)	51 (89.5)	0.582
≥5000 copies/ml	12 (5.6)	8 (9.2)		6 (5.3)	5 (8.8)	

IC + CCRT, induction chemotherapy plus concurrent chemoradiotherapy; CCRT, concurrent chemoradiotherapy.

### Survival outcomes according to treatment modality

With a median follow-up of 41 months (range, 7–90 months), the survival outcomes of IC + CCRT were superior to those of CCRT. The 3-year LRFS, DMFS, PFS and OS were 96.6%, 81%, 80.7% and 84.7% for IC + CCRT and 90.1%, 71.1%, 62% and 75.6% for CCRT, respectively (LRFS, p < 0.001; DFMS, p = 0.056; PFS, p < 0.001; OS, p = 0.007; Table 2). On propensity matched survival analysis, there was no significant difference between two treatment groups for 3-year LRFS (p = 0.65), however, IC + CCRT showed significantly better outcomes than CCRT in 3-year DMFS, PFS and OS (DFMS, p = 0.045; PFS, p < 0.001; OS, p = 0.011 Table 2).

**Table 2:** 3-year survival outcomes for patients received different treatment model.

	Before match		P value	After match		P value
	IC + CCRT	CCRT		IC + CCRT	CCRT	
LRFS	96.6%	90.1%	<0.001	96.9%	100%	0.65
PFS	80.7%	62%	<0.001	78.1%	56%	<0.001
DMFS	81%	71.1%	0.056	82%	66.5%	0.045
OS	84.7%	75.6%	0.007	83.8%	72.7%	0.011

IC + CCRT, induction chemotherapy plus concurrent chemoradiotherapy; CCRT, concurrent chemoradiotherapy; LCFS, local recurrence-free survival; PFS, progression free survival; DMFS, distant metastasis-free survival; OS, overall survival.

### Prognostic analysis

On univariate analysis, WHO histological type, N stage, treatment modality were associated with DMFS and OS (Table 3). Multivariate analysis showed that WHO histological type, N stage, treatment modality were independently prognostic factors for DMFS (WHO histological type, hazard ratio (HR) 1.845, 95% confidence interval (95% CI) 1.18–3.047, p = 0.017; N stage, HR 1.916, 95% CI 1.35–2.717, p < 0001; treatment modality, HR 2.525, 95% CI 1.469–4.34, p = 0.001) and OS (WHO histological type, HR 1.982, 95% CI 1.204–3.262, p = 0.007; N stage, HR 1.824, 95% CI 1.317–2.526, p < 0001; treatment modality, HR 2.852, 95% CI 1.68–4.844, p < 0.001) (Table 4).

**Table 3:** Univariate analysis of prognostic factors for all patients.

	DMFS		OS	
	HR (95% CI)	P value	HR (95% CI)	P value
Age (>50 vs. ≤50)	1.191 (0.725–1.956)	0.49	1.309 (0.799–2.145)	0.286
Gender (male vs. female)	1.112 (0.632–1.959)	0.712	1.02 (0.572–1.819)	0.946
Smoking (yes vs. no)	1.072 (0.657–1.751)	0.78	0.814 (0.497–1.335)	0.415
Drinking (yes vs. no)	1.048 (0.629–1.746)	0.858	0.779 (0.455–1.332)	0.362
Race (ethnic Han vs. other)	1.973 (0.619–6.295)	0.251	1.956 (0.613–6.237)	0.257
EBV copies (≥5000 vs. <5000)	0.42 (0.103–1.718)	0.228	0.914 (0.332–2.515)	0.862
WHO type (IIa vs. IIb)	1.757 (1.041–2.852)	0.034	1.943 (1.185–3.185)	0008
T stage (T3-4 vs. T1-2)	0.964 (0.754–1.232)	0.768	1.199 (0.922–1.559)	0.176
N stage (N2-3 vs. N0-1)	1.599 (1.13–2.263)	0.008	1.402 (1.004–1.958)	0.048
Clinical stage (IV vs. III)	1.521 (0.84–2.754)	0.166	1.986 (1.058–3.728)	0.033
Treatment modality (CCRT vs. IC + CCRT)	1.628 (1.008–2.707)	0.048	1.956 (1.191–3.214)	0.008
Tumor volume (continuous variable)	1.081 (0.868–1.346)	0.488	1.328 (1.062–1.661)	0.013

**Table 4:** Multivariate analysis of prognostic factors for all patients.

	DMFS		OS	
	HR (95% CI)	P value	HR (95% CI)	P value
WHO type (IIa vs. IIb)	1.845 (1.118–3.047)	0.017	1.982 (1.317–2.526)	0007
N stage (N2-3 vs. N0-1)	1.916 (1.35–2.717)	<0.001	1.824 (1.317–2.526)	<0.001
Treatment modality (CCRT vs. IC + CCRT)	2.525 (1.469–4.34)	0.001	2.852 (1.68–4.844)	<0.001
Tumor volume (continuous variable)	—	—	1.342 (1.07–1.682)	0.011

DMFS, distant metastasis-free survival; OS, overall survival.

### Efficacy of IC + CCRT for patients stratified as WHO type IIa and WHO type IIb

The stratified analysis was conducted to further evaluate the efficacy of IC + CCRT in different WHO types. The clinical characteristics of treatment groups were well balanced in different nonkeratinizing carcinoma subtypes (Tables A1 and 2).

**Figure 1:**
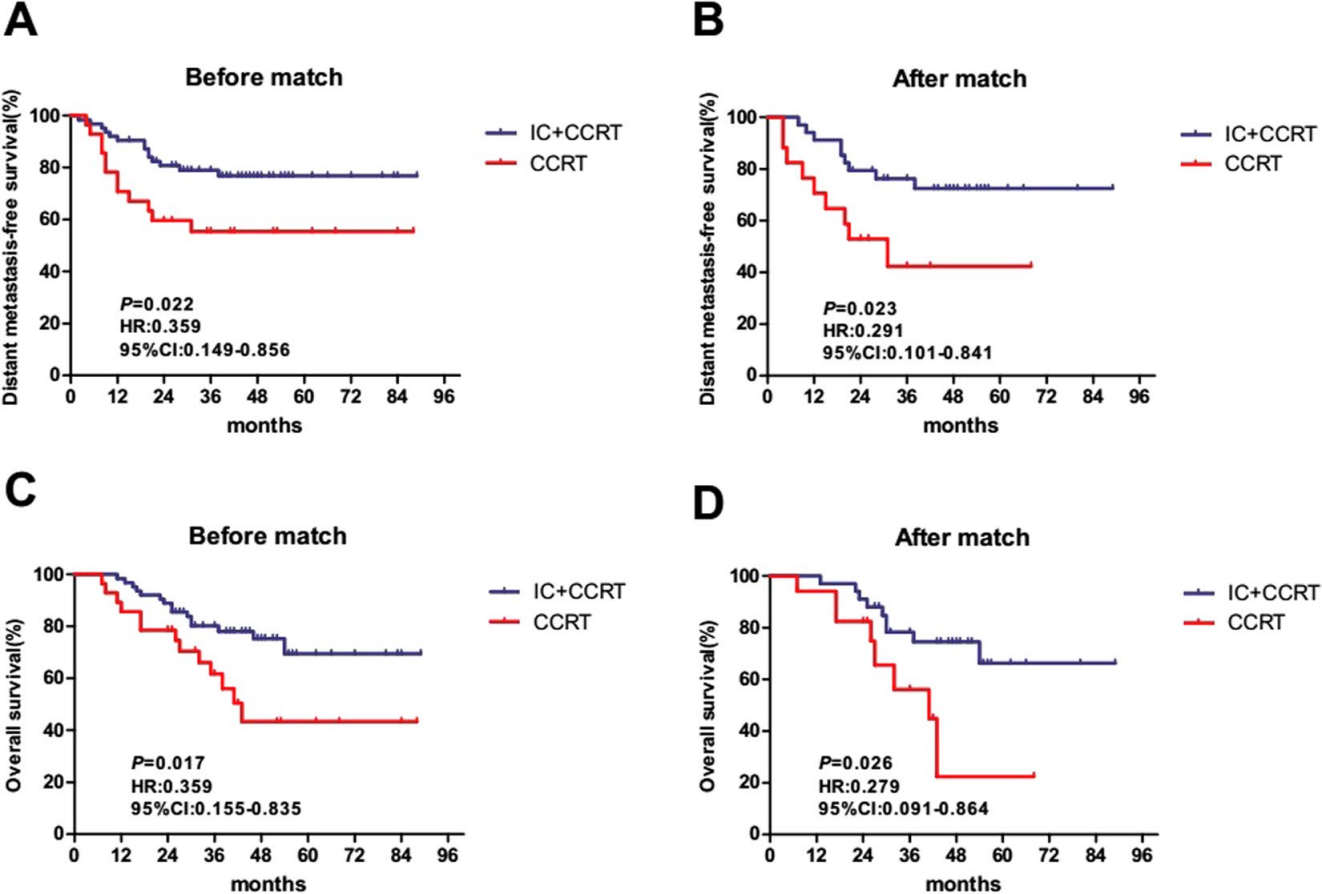
Before and after propensity matching, Kaplan-Meier DMFS and OS curves for the two treatment group in patients with WHO histological type IIa. (**A**,**B**) Distant metastasis-free survival; (**C**,**D**) overall survival; IC + CCRT, induction chemotherapy plus concurrent chemoradiotherapy; CCRT, concurrent chemoradiotherapy.

**Figure 2:**
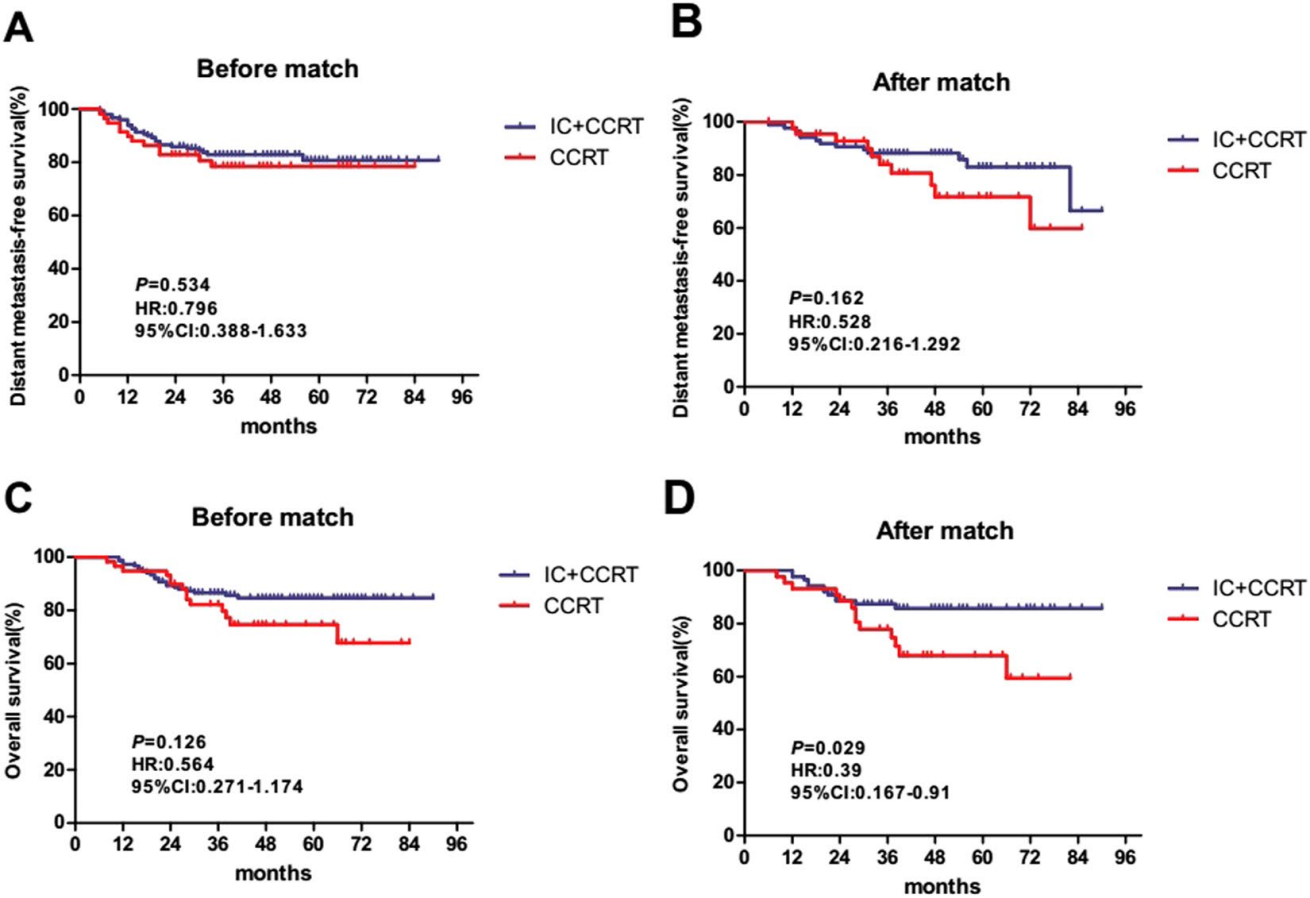
Before and after propensity matching, Kaplan-Meier DMFS and OS curves for the two treatment group in patients with WHO histological type IIb. (**A**,**B**) Distant metastasis-free survival; (**C**,**D**) overall survival; IC + CCRT, induction chemotherapy plus concurrent chemoradiotherapy; CCRT, concurrent chemoradiotherapy.

For the WHO type IIa cohort, a propensity matched analysis was conducted with 1:1 matching (18 IC + CCRT patients with 18 CCRT patients). IC + CCRT showed a better DMFS and OS than CCRT (DMFS, HR: 0.221, 95% CI: 0.072–0677, p = 0.0082; OS, HR: 0.147, 95% CI: 0.046–0.476, p = 0.0014) (Supplementary Figure). To improve the statistical power, another propensity matched analysis was conducted with 2:1 matching (34 IC + CCRT patients with 17 CCRT patients). IC + CCRT was also found to produce better 3-year DMFS and OS than CCRT (DMFS, 76.2% vs. 42.2%, p = 0.029; OS, 78.3% vs. 65.5%, p = 0.027) (Fig. 1). For the WHO type IIb cohort, IC + CCRT showed a better trend for DMFS (HR: 0.397, 95% CI: 0.147–1.067, p = 0.067) and a statistically significant improvement for OS (HR: 0.241, 95% CI: 0.089–0.647, p = 0.0047) compared with CCRT based on the 1:1 propensity matching (54 IC + CCRT patients with 54 CCRT patients) (Supplementary Figure). On propensity matched with 2:1 matching (88 IC + CCRT patients, 44 CCRT patients), there was a trend of better 3-year DMFS for IC + CCRT compared with CCRT (85.9% vs. 76%, p = 0.162), but a statistically significant difference was showed between IC + CCRT and CCRT for 3-year OS (87.4% vs. 77.9%, p = 0.029) (Fig. 2).

## Discussion

To the best of our knowledge, this is a first study to detect the efficacy of IC + CCRT in patients of NPC with different non-keratinizing subtypes. According to the results of our study, IC + CCRT had better efficacy to increase the OS compared with CCRT in patients with WHO type IIb. Moreover, IC + CCRT had a superior efficacy to improve OS and DMFS for patients with WHO type IIa compared with CCRT.

Recently, a randomized trial from endemic region of China indicated IC + CCRT produced a excellent 3-year DMFS (90%) and OS (92%) for locoregionally advanced NPC compared with CCRT (83% for DMFS, 86% for OS)^14^. Our previous experience of using IC + CCRT in locaregionaly advanced NPC in non-endemic of China confirmed the promising efficacy of IC + CCRT. The 3-year local control, DMFS and OS achieved 94.9%, 78.6% and 84.5%, respectively^7^. However, our previous study could not answer this question that whether IC + CCRT could provide any additional survival benefit compared with standard CCRT for non-endemic patients. In this propensity matched study, we reported IC + CCRT produced superior survival outcomes than CCRT for patients from northwest of China. Although the results of this study had similar outcomes to reports from endemic data of China, the survival data of IC + CCRT were lower than the endemic data^14,17,18^. It may be explained by three reasons: (1) this study included higher proportion of stage IV patients (approximately 70%) than ever reports from endemic data; (2) high proportion of WHO type IIa contributed the slightly worse outcomes; (3) a few of patients received PF (4.7%, data was not shown) as induction chemotherapy regimens, which was indicated as a worse option than other regimens contained docetaxel and gemcitabine in NPC^7,14,19^.

A few of studies have revealed worse prognosis in keratinizing squamous carcinoma of the nasopharynx compared with the non-keratinizing category in non-endemic and endemic areas^3,20–22^. Similarly, for patients with non-keratinizing categories of NPC, differentiated subtype generated worse prognosis than undifferentiated subtype. Cheng *et al*. reported that WHO type IIa contribute a worse locoregional control due to radioresistance^2^. Another study also found patients with WHO type IIa contributed to a worse DMFS and OS than patients with WHO type IIb^8^. Our previous study also found that WHO type IIa was an independently poor prognostic factor for DMFS and OS in patients from northwest of China^5^. These studies above mentioned implied that patients with WHO type IIa might be received more intensity treatment modalities. In the subgroup propensity matched analysis of this study, IC + CCRT demonstrated better survival outcomes than CCRT in patients with WHO types IIb. The study could offer a reference for clinicians that locoregionally advanced NPC patients with WHO type IIa could obtained survival benefit from IC + CCRT but CCRT. However, the results should also be understood carefully because the samples size of WHO type IIa was too small to have enough power to obtain a reliable conclusion. Prospective studies with large cohort should be designed to investigate the role of IC + CCRT in patients with WHO type IIa of NPC.

The mechanism of WHO type IIa leading to the poor prognosis remains unclear. Some studies reported high expression of IKK-αcould induce NPC cell differentiation via regulating the NF-κB signaling pathway^23–25^. Others reported expression of ERCC1, which was relative to cisplatin-based chemotherapy resistance in cancers, was higher in the WHO type IIa compared with WHO type IIb^8,26^. These studies implied that WHO type IIa had distinct characteristics of molecular biology, and the treatment for this subtype should focus on inducing the differentiation of NPC cells via regulating the NF-κB signaling pathway or decreasing the internal chemotherapy-resistance molecular to increase the treatment sensitivity in the future.

According to the subgroup analysis for patients with WHO type IIc, IC + CCRT could significantly improve OS compared with CCRT (3-year OS, IC + CCRT 87.4% vs. CCRT 77.9%). This result was similar to endemic data. However, only better trend but significant improvement was detected for DMFS when IC + CCRT compared with CCRT after propensity matching. Maybe a positive result would be possible to get by a longer follow-up time.

EBV DNA copies were well established as an independently prognostic factor for NPC outcomes. In endemic region of China, EBV DNA was detected in nearly 90% patients, which was significantly higher than the detection rate (only approximate 10%) in this study and our previous studies^7,15^. Due to the small number of patients with EBV DNA positivity, we could not analyze any correlation between EBV and prognosis in this study. Maybe it was not reasonable to define 5000 copies as cut-off value in this study because of lack of verification by the receiver operating characteristic curves. However, the reported cut-off value of EBV DNA load was variable in many studies, even variable EBV cut-off value were reported in same center^17,27^. Overall, the proportions of more than 5000 copies in our center were lower than the reports of endemic region^17,27,28^. The potential reasons for this situation might be explained as follows: (1) lack of unified methods for the detection of EBV DNA; (2) low copies of EBV DNA in non-endemic NPC; (3) specific infection status of EBV in non-endemic region of China; (4) presence of specific EBV strain infection in non-endemic region of China.

## Conclusion

Compared with CCRT, IC + CCRT could improve the distant metastasis-free survival and overall survival in advanced stage NPC patients with different nonkeratinizing carcinoma subtypes. However, prospective studies with large cohort are needed to further assess the eventually efficacy of IC + CCRT for patients with WHO type IIa.

## Electronic supplementary material

dataset 1
