# Supplementary material for: Induction chemotherapy followed by concurrent chemoradiotherapy is benefit for advanced stage nasopharyngeal carcinoma with different nonkeratinizing carcinoma subtypes
Source: Sci Rep. 2018 Sep 6;8:13318. doi: 10.1038/s41598-018-31050-z (PMC6127191; doi:10.1038/s41598-018-31050-z)
Supplement: Supplementary file 1 — dataset 1 [file 41598_2018_31050_MOESM1_ESM.pdf]

Induction chemotherapy followed by concurrent chemoradiotherapy is benefit for advanced stage nasopharyngeal carcinoma with different nonkeratinizing carcinoma subtypes

Jian Zang [a†], Chen Li [b†], Man Xu [a], Wanni Xu[c], Xiaowei Kang[d], Jianhua Wang[a], Shanquan Luo[a], Mei Shi[a*]

Table A1 clinical characteristics of patients with WHO type IIa before and after PSM by treatment

	Before match			After match		
	IC+CCRT N(%)	CCRT N(%)	P value	IC+CCRT	CCRT	P value
Gender						
Male	48(76.2)	20(71.4)	0.63	29(85.3)	13(76.5)	0.436
female	15(23.8)	8(28.6)		5(14.7)	4(23.5)	
age						
≤50	36(57.1)	14(50)	0.527	15(44.1)	8(47.1)	0.842
>50	27(42.9)	14(50)		19(55.9)	9(52.9)	
Race						
Ethnic Han	63(100)	28(100)	-	34(100)	17(100)	-
others	0(0)	0(0)		0(0)	0(0)	
Smoke						
No	32(50.8)	16(57.1)	0.576	16(47.1)	8(47.1)	1.000
yes	31(49.2)	12(42.9)		18(52.9)	9(52.9)	
Smoke index						0.552
mean	263.33±49.39	200.36±56.99	0.452	236.18±56.25	300.58±85.62	
Drink						
No	39(61.9)	20(71.4)	0.38	17(50)	9(52.9)	0.843
Yes	24(38.1)	8(28.6)		17(50)	8(47.1)	
AJCC stage						
III	18(28.6)	4(14.3)	0.142	11(32.4)	2(11.8)	0.112
IV	45(71.4)	24(85.7)		23(67.6)	15(88.2)	
T stage						
T1	2(3.2)	0(0)	0.016	0(0)	0(0)	0.268
T2	20(31.7)	2(7.1)		12(35.3)	3(17.6)	
T3	13(20.6)	4(14.3)		4(11.8)	1(5.9)	
T4	28(44.4)	22(78.6)		18(52.9)	13(76.5)	
N stage						
N0	3(4.8)	13(46.4)	<0.001	0(0)	0(0)	1.000
N1	4(6.3)	3(10.7)		2(5.9)	1(5.9)	
N2	36(57.1)	10(35.7)		28(82.4)	14(82.4)	
N3	20(31.7)	2(7.1)		4(11.8)	2(11.8)	
Tumor volume ml						
Median(range)	43.2(12.5-118.4)	50.1(20.9-105.25)		45.1(15.5-109.1)	36.7(20.9-106.5)	
<23.75	10(15.9)	1(3.6)	0.327	3(8.8)	1(5.9)	0.887

23.75-42.14	21(33.3)	9(32.1)		13(38.2)	8(47.1)	
42.15-64.67	17(27)	8(28.6)		11(32.4)	4(23.5)	
>64.67	15(23.8)	10(35.7)		7(20.6)	4(23.5)	
EBV DNA						
<5000 copies/ml	59(93.7)	27(96.4)	0.591	30(88.2)	16(94.1)	0.505
≥5000 copies/ml	4(6.3)	1(3.6)		4(11.8)	1(5.9)	

Table A2 clinical characteristics of patients with WHO type IIb before and after PSM by treatment

	Before match			After match		
	IC+CCRT N(%)	CCRT N(%)	P value	IC+CCRT	CCRT	P value
Gender						
Male	116(76.8)	4(79.7)	0.657	72(81.8)	35(79.5)	0.753
female	35(23.2)	12(20.3)		16(18.2)	9(20.5)	
age						
≤50	99(65.6)	34(57.6)	0.283	60(68.2)	29(65.9)	0.793
>50	52(34.4)	25(42.4)		28(31.8)	15(34.1)	
Race						
Ethnic Han	144(95.4)	58(98.3)	0.317	83(94.3)	43(97.7)	0.375
others	7(4.6)	1(1.7)		5(5.7)	1(2.3)	
Smoke						
No	72(47.7)	35(59.3)	0.129	50(56.8)	26(59.1)	0.803
yes	79(52.3)	24(40.7)		38(43.2)	18(40.9)	
Smoke index						
mean	255.26±28.97	212.03±46.97	0.431	189.86±33.74	173.96±44.38	0.78
Drink						
No	95(62.9)	42(71.2)	0.258	63(71.6)	31(70.5)	0.892
Yes	56(37.1)	17(28.8)		25(28.4)	13(29.5)	
AJCC stage						
III	39(25.8)	23(39)	0.06	31(35.2)	19(43.2)	0.374
IV	112(74.2)	36(61)		57(64.8)	25(56.8)	
T stage						
T1	9(6)	6(10.2)	0.259	7(8)	6(13.6)	0.752
T2	43(28.5)	10(16.9)		24(27.3)	10(22.7)	
T3	28(18.5)	10(16.9)		12(13.6)	6(13.6)	
T4	71(47)	33(55.9)		45(51.1)	22(50)	
N stage						
N0	2(1.3)	9(15.3)	<0.001	0(0)	0(0)	1.000
N1	17(11.3)	10(16.9)		7(8)	5(11.4)	
N2	96(63.6)	37(62.7)		76(86.4)	36(81.8)	
N3	36(23.8)	3(5.1)		5(5.7)	3(6.8)	
Tumor volume						

ml						
Median(range)	40.27(16.5-224.1)	42.1(17.8-135.6)		40.03(18.2-189.4)	42.6(17.3-135.6)	
<23.75	48(31.8)	16(27.1)	0.408	31(35.2)	14(31.8)	0.493
23.75-42.14	32(21.2)	14(23.7)		16(18.2)	8(18.2)	
42.15-64.67	32(21.2)	18(30.5)		20(22.7)	15(34.1)	
>64.67	39(25.8)	11(18.6)		21(23.9)	7(15.9)	
EBV DNA						
<5000 copies/ml	143(94.7)	52(88.1)	0.097	83(94.3)	40(90.9)	0.464
⩾5000 copies/ml	8(5.3)	7(11.9)		5(5.7)	4(9.1)	

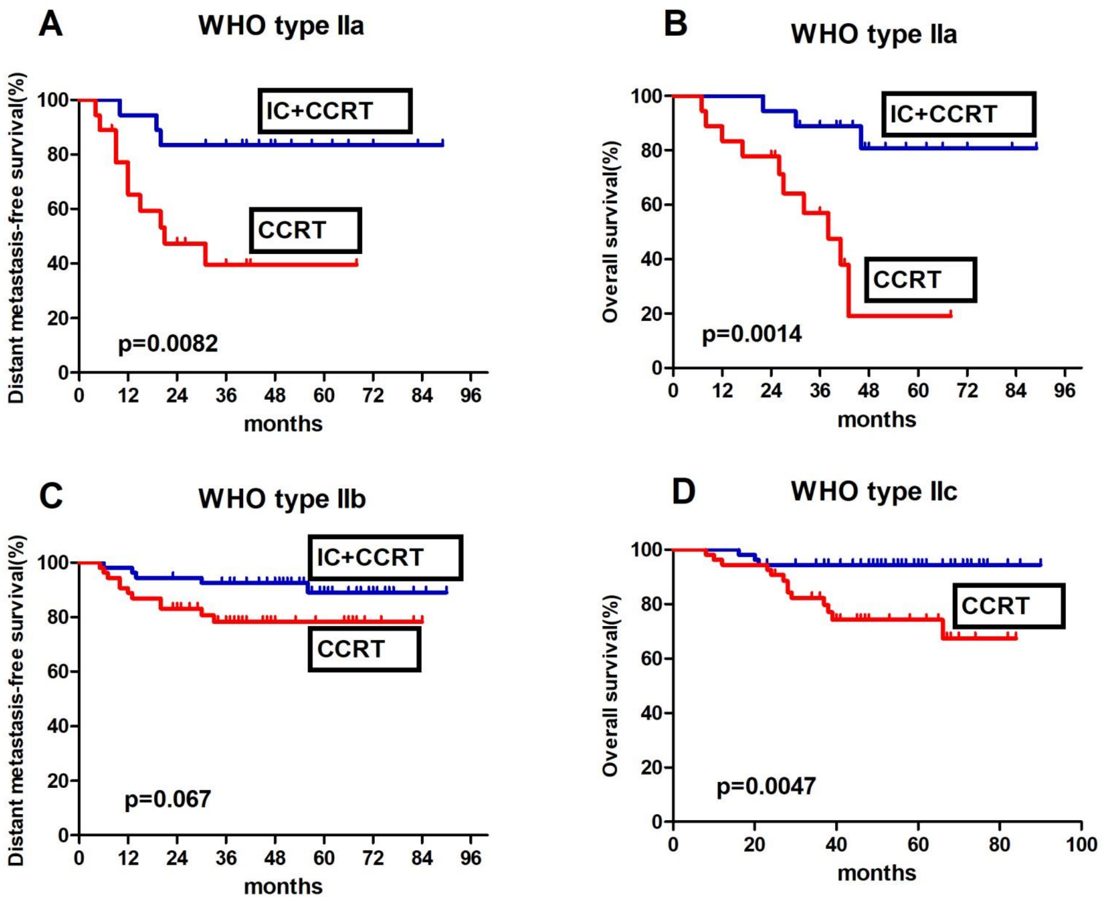

Supplementary figure legend

After propensity matching with 1:1, Kaplan-Meier DMFS and OS curves for the two treatment group in patients with different nonkeratinizing carcinoma. (A) and (C), distant metastasis-free survival; (B) and (D), overall survival; IC+CCRT, induction chemotherapy plus concurrent chemoradiotherapy; CCRT, concurrent chemoradiotherapy.
